# A retrospective analysis of cannabis use in a cohort of mentally ill patients in Sri Lanka and its implications on policy development

**DOI:** 10.1186/1747-597X-5-16

**Published:** 2010-07-08

**Authors:** Chaturaka Rodrigo, Srina Welgama, Alwis Gunawardana, Chinthaka Maithripala, Gamini Jayananda, Senaka Rajapakse

**Affiliations:** 1 Psychiatry Unit, Provincial General Hospital, Ratnapura, Sri Lanka; 2 Department of Clinical Medicine, Faculty of Medicine, University of Colombo, Sri Lanka

## Abstract

**Background:**

Several epidemiological studies have shown that cannabis; the most widely used illegal drug in the world, is associated with schizophrenia spectrum disorders (SSD).

**Aims:**

To assess the characteristics of cannabis use and its association with SSD in a cohort of psychiatrically ill patients and discuss the implications for policy development

**Methods:**

This is a retrospective analytical study of a cohort of psychiatric patients who received treatment in the psychiatry unit of the Provincial General Hospital, Ratnapura, Sri Lanka over five years (2000 - 2004). The schizophrenia spectrum disorders defined in this article include schizophrenia and the schizoaffective disorders.

**Results:**

A total of 3644 patient records were analyzed. The percentage of self reported life time cannabis (LTC) use was 2.83% (103, all males). Sixteen percent (576) of the total cohort was diagnosed with SSD by 2009. Male sex and LTC use were significantly associated with SSD (p < 0.01 and 0.001 respectively). In the majority (91.5%), cannabis use preceded the diagnosis. There were 17(16.5%) patients diagnosed as cannabis induced psychosis and 7 (41.2%) of them were subsequently diagnosed as SSD. This group was significantly more likely to have had a past psychiatric consultation, but other demographic and clinical correlates did not differ from the rest of the LTC users.

**Conclusions:**

Self reported LTC use was strongly associated with being diagnosed with SSD. However we could not identify a particular subgroup of users that are at increased risk to recommend targeted primary prophylaxis. The policy implications of this observation are discussed.

## Background

The plant *Cannabis sativa *grows in temperate and tropical climates. Its seeds, flowering tops, leaves and stalks contain a cocktail of chemicals termed cannabinoids, some of which (e.g.- Δ^9 ^tetrahydrocannabinol) cause psychoactive manifestations following ingestion or inhalation of smoke[1]. It is considered to be the most widely used illegal drug in the world and 4% of world's adult population (160 million) were using cannabis in 2005[2]. This was a 10% increase compared to the estimates in 1995.

The evidence for an association between cannabis use and schizophrenia spectrum disorders (SSD) was demonstrated more than 20 years ago [3-5]. The first landmark longitudinal study demonstrating such a link was published in 1987 by Andreasson et al[3]. They showed (in a 15 year follow up of 45,570 Swedish conscripts) a higher risk of schizophrenia among heavy cannabis users [Relative risk (RR) - 6.0]. A follow up study of 535 patients treated for cannabis induced psychotic symptoms in Denmark observed that 238 (44.5%) of them were subsequently treated for schizophrenia spectrum disorders. Male gender and younger age were associated with an increased risk for SSD[6]. Arseneault et al[7] have also observed a higher risk of schizophrenia in young cannabis users. They analyzed data from a birth cohort in Dunedin, New Zealand. Younger age of cannabis use (age 15 vs. 18) was associated with a greater risk of schizophrenia and the cannabis users were more likely to be symptomatic by age 26 than controls. Still, a majority of users did not develop psychotic symptoms. Barnes et al[8] have also shown that male gender and cannabis use were significantly associated with subsequent schizophrenia and a younger age of onset of illness. They further showed that such an association did not exist for alcohol or any other substance examined (hallucinogens, stimulants, opiates).

While such observations are made repeatedly, some questions are left unanswered. One is regarding the actual mechanism of the association between cannabis and SSD. Different theories have been proposed each having its proponents and opponents. They are as follows: a) cannabis use causes SSD (causal model); b) patients with SSD are attracted to cannabis use; c) cannabis use induces SSD in a minority with background vulnerability [9,10]. The theory of background vulnerability (third hypothesis) has been a point of interest in recent research [11]. However, the exact nature of this vulnerability is unclear (e.g. genetic, environmental or a combination of them). On the genetic aspect, a functional polymorphism of the gene encoding the catechol-O-methyl transferase (COMT) enzyme (which degrades catecholamines such as dopamine, noradrenaline etc.) has been of interest. The polymorphism results in two alleles of high (Val) and low (Met) enzymatic activity. The highly active variant is hypothesized to cause imbalance in dopamine concentrations in prefrontal and mesolimbic areas resulting in cognitive defects and psychotic phenomena respectively. One study has shown that those homozygous for Val gene or even carriers of it are more likely to have psychotic symptoms and a subsequent diagnosis of a SSD following cannabis use [11]. The influence of environmental factors may also be relevant but the evidence is limited. Compton et al [12,13] have demonstrated that childhood experiences of physical and sexual abuse were significantly more in patients with a dual diagnosis of first episode SSD and cannabis use. However the sample size in this study was small to come to a valid conclusion.

Another interesting hypothesis that contrasts the theories supporting an aetiological role of substance use on schizophrenia needs to be mentioned as well. This theory was formulated by the repeated observation of a better premorbid adjustment (less social withdrawal, better peer relations, socio sexual adjustment and interests) in substance users and abusers[14]. It was proposed that people with better premorbid adjustment have an increased risk of exposure to substance use due to their sociability. Later, when some of them develop schizophrenia (by an unrelated process), the stresses of illness may drive them to have pronounced substance abuse as a maladaptive coping strategy. Yet an observer (to whom early features of prodrome are not obvious but the increase in substance use is) may think that heavy substance use preceded and hence had an aetiological role in schizophrenia. The initial studies showing a better pre morbid adjustment in substance users with psychiatric illnesses did not differentiate between the substances used[15,16]. However Arendt et al[14] have demonstrated a statistically significant better premorbid adjustment among exclusive alcohol abusers and cannabis abusers with schizophrenia. Still, the number of exclusive cannabis abusers was small (n-9) in this cohort. Another fact that favoured this hypothesis was the absence of a similar observation among stimulant and hallucinogen abusers and non abusers. The impact of these substances on neurotransmitter systems had been demonstrated and it was assumed that the lack of such an association was due to their (hallucinogens and stimulants) possible aetiological role in psychosis. However since the publication of these observations in 1992, the impact of cannabinoids on neurotransmitter systems has also been speculated, backed by the findings of several experimental neurobiological studies. The current opinion is that modulation of release of neurotransmitters; dopamine, gamma amino-butyric acid (GABA) and glutamate by cannabinoids (acting via the cannabinoid receptor 1 in mesolimbic and mesocortical systems) account for its psychotic manifestations[10]. There is also heterogeneity and contrasting effects of various cannabinoids in this regard. For example, tetrahydrocannabinol (THC) has mainly psychomimetic and anxiety enhancing effects while cannabidiol (another cannabinoid compound) is shown to have anxiolytic and antipsychotic properties [17-19].

Another grey area is the diagnosis of 'cannabis induced psychosis' (CIP) which is sometimes used to refer to the psychotic manifestations with cannabis. Experimental studies with THC have shown it to induce both positive and negative symptoms with cognitive deficits in both healthy individuals and schizophrenic patients [20,21]. However, several clinical studies have shown that the patients with 'cannabis induced psychosis' and those subsequently diagnosed with schizophrenia demonstrate more positive symptoms and fewer negative symptoms [10,22-26]. Some authors doubt the diagnosis of CIP altogether. They argue that it is early onset schizophrenia [27,28]

Most of the research on cannabis use and its sequelae are from cohorts in developed countries. There are only a few recent studies on cannabis use in the South Asian region and this appears to be the first analytical study on cannabis use among psychiatric patients in Sri Lanka.

Based on the above observations, this study was designed to answer several queries;

a) to identify the characteristics of cannabis use in this sample

b) to confirm the existence of an association between cannabis use and subsequent development of SSD in this sample.

c) in the event of finding such an association, to explore the evidence with relation to the existing theories on cannabis and SSD.

d) to characterize the impact of environmental and other confounding factors on cannabis use and SSD

e) to explore whether CIP patients as a group differed from the rest of the cannabis users to justify the uniqueness of this diagnosis

f) to recommend policy changes on cannabis use based on the findings of the study

## Methods

This is a retrospective analytical study of a cohort of psychiatrically ill patients who received treatment in the Provincial General Hospital, Ratnapura (PGHR), Sri Lanka over the five years from 2000 Jan 1^st ^to 2004 Dec 31^st^. The PGHR and its psychiatry unit cater to the healthcare needs of approximately 9.4% (1.8 million in 2003) of the country's population in both urban and rural areas [29]. Furthermore, it was the only center in the district with inpatient treatment facilities under the supervision of a consultant psychiatrist during the time period for this retrospective follow up (2000 - 2004). Therefore it is likely that a majority of patients with psychiatric symptoms came to this unit for treatment as it was the only option available. The records were analyzed in 2009. At this point we were able to obtain data over a follow up period of between 5 and 9 years.

The investigators examined the records of all patients treated within the specified time period in the psychiatry unit of PGHR. Each patient seen at clinic or ward is allocated a separate file with a clinic number and all the data on this patient (clinic visits or admissions) are updated in the same file over the years. Every patient admitted (or assessed in the clinic) is seen by two doctors (first assessed by the attending doctor of the unit and later reviewed by the consultant psychiatrist). A detailed history is recorded on the first page of the patient's records. The same procedure is repeated with each subsequent admission. Details on psychoactive substance use are queried from patients as a routine on every admission and documented. This includes the use of alcohol, nicotine and any other common substances of abuse. However laboratory facilities were not available at that time to test for serum or urinary levels of narcotics in Ratnapura (routine or 'on request' testing). Even now, such facilities are not available and if urine needs to be tested for cannabinoids, a sample has to be sent to the capital Colombo to be tested in a private laboratory at patient's expense. Therefore, the substance use documented in records was obtained by self report only. The logistical difficulties mentioned above prevented independent laboratory confirmation of substance use.

All the patient records from 2000 to 2004 were examined for a history of life time cannabis use and the data of these patients were extracted to a data sheet. The term life time cannabis (LTC) use in this paper strictly refers to self reported LTC use. Data were collected on demography, clinical presentation, cannabis use, concurrent substance use, treatment and follow up details for those reporting LTC use.

These data were analyzed and compared in several ways to achieve the objectives cited above. The analysis was arranged to;

a) compare the prevalence of schizophrenia spectrum disorders between the life time cannabis (LTC) user group and the non users

b) Analyze the demographic and clinical profile of LTC users

c) Comparison of SSD and non SSD patients in LTC user group to identify any characteristic features of the earlier subgroup

d) Try to establish any unique characteristics of the group diagnosed as CIP by

i. comparing them with other LTC users

ii. comparing the CIP patients diagnosed with SSD with other SSD patients in the LTC user group

e) To summarize the findings to identify an association between cannabis use and subsequent development of SSD

A clear establishment of the timeline between cannabis use, onset of psychotic symptoms and diagnosis of SSD was important to come to valid conclusions. All the patient records were dated before each entry by the attending doctors so there was no confusion in establishing the timeline after the patient was registered in the unit. The date of diagnosis of SSD was established as the date such a diagnosis was first documented in the patient records provided there were concurrent documentation of symptoms keeping with the ICD - 10 (International Classification of Diseases - 10) diagnostic criteria. The data extraction sheet had a checklist referring to each diagnostic criteria of ICD -10 for this purpose. Following the establishment of the date of diagnosis, any recorded cannabis use before and after (as mentioned in records) were noted. However, the formal date of diagnosis does not necessarily indicate the actual onset of disease. The 'onset' of disease was difficult to establish from clinic records alone due to missing data and the retrospective nature of the study. Therefore, we were restricted to establish the timeline between cannabis use and the diagnosis of illness (easier to establish from records) rather than between cannabis use and the 'onset of illness' (difficult to establish from records).

The data were analyzed with SPSS v15 statistical software, and significance of associations were calculated using appropriate statistical tests such as chi square test and Fisher's exact test. Findings relevant to descriptive statistics were summarized into proportions and averages (mean/standard deviation or percentiles) based on the scales of measurements. Comparison of means was done with unpaired t test. Ethical clearance for the study was granted by the ethics review committee of Faculty of Medicine, University of Colombo.

The schizophrenia spectrum disorders identified in this article include all classifications of schizophrenia and schizoaffective disorders. There were no diagnoses in the LTC user cohort for schizotypal and schizoid personality disorders. ICD - 10 is the manual used for diagnosis in Sri Lankan psychiatry units.

## Results

Three thousand six hundred and forty four (3644) patient records (males - 2095, females - 1549) were scrutinized for evidence of cannabis use. Evidence of LTC use was found in 103 (2.83%) patients. All patients with a history of cannabis use were males.

The majority of LTC users belonged to the 21 - 30 age group (47, 45.6%) and the Sinhalese ethnicity (95, 92.2%). Fifty four (52.4%) admitted to concurrent alcohol use and 21 (20.4%) to nicotine use. One person had used heroin. The socio-demographic variables of LTC users are summarized in table 1.

**Table 1:** Descriptive statistics of the socio-demographic variables of patients with life time cannabis (LTC) use

Variable	Number	Percentage (%)
**Sex**		

Male	103	100

Female	0	0

**Age (years)**		

0 - 10	0	0.0

11 - 20	10	9.7

21 - 30	47	45.6

31 - 40	22	21.4

41 - 50	9	8.7

51 - 60	6	5.8

61 - 70	1	1.0

Unknown	8	7.8

**Civil status**		

Married	31	30.1

Unmarried	46	44.7

Unknown	26	25.2

**Level of education**		

No formal education	4	3.9

Primary education	31	30.1

Secondary education	44	42.7

Tertiary education	0	0.0

Unknown	24	23.3

**Ethnicity**		

Sinhala	95	92.2

Tamil	6	5.8

Muslim	2	1.9

**Concurrent substance use**		

Alcohol*	54	52.4

Nicotine*	21	20.4

Heroin	1	1.0

Other	0	0.0

**Past history of a psychiatric consultation**		

Yes	42	40.8

No	61	59.2

**Family history of a psychiatric disorder**		

Yes	18	17.5

No	85	82.5

*Data on alcohol use was missing in 2 patients and that of nicotine use was missing in 13 patients.

The results are presented under the following subtopics

1. Comparison between LTC user SSD patients and non-cannabis-using SSD patients

2. Characteristics of cannabis use in the sample

3. Comparison between SSD patients and non-SSD patients in the LTC user group

4. Comparison between patients diagnosed as cannabis induced psychosis (CIP) and the rest of the LTC user group

5. Comparison between SSD patients in CIP (CIP-SSD) group and other cannabis using SSD patients

### Comparison between LTC user- SSD patients and non cannabis using SSD patients

Of the total sample, 576 (15.8%) patients were diagnosed with a schizophrenia spectrum disorder (SSD) by 2009 [males: 359 (62.3%), females: 217 (37.7%)]. Males were more likely to be diagnosed with a SSD [RR: 1.22, 95% confidence interval (CI): 1.04-1.42] (chi square test, df - 1, χ^2 ^- 6.54, p-0.01). In the LTC user group, 47 (45.6%, all males) were diagnosed with SSD during follow up (by 2009). Of them, 43 were diagnosed with schizophrenia and 4 with schizoaffective disorder. This was 8.1% of the total sample with SSD and 13.1% of the males with SSD (table 2). The relative risk of a LTC user to have SSD was 3.05 (95% CI: 2.44- 3.82) compared to a non user and when males alone were considered, it was 2.91 (95% CI: 2.31- 3.68). Both these associations were statistically significant (chi square test, df - 1, χ^2 ^- 70.58, p < 0.0001), (chi square test, df - 1, χ^2 ^- 80.01, p < 0.0001).

**Table 2:** Number of patient records analyzed according to sex, cannabis use and presence of schizophrenia spectrum disorders (SSD) [total (n) - 3644]

Year	Male				Female (all NCU)	
	
	SSD present		SSD absent		SSD present	SSD absent
	
	LTC Users*	NCU^†^	LTC Users*	NCU^†^		
2004	16	44	12	312	38	296

2003	9	79	15	399	39	309

2002	6	61	7	400	48	312

2001	8	73	12	330	44	252

2000	8	55	10	239	48	163

**Total**	**47 (1.3)**	**312 (8.6)**	**56 (1.5)**	**1680 (46.1)**	**217 (6.0)**	**1332 (36.6)**

*LTCU -life time cannabis users, ^†^NCU - Non cannabis users. The percentages are given within brackets in the totals row

### Characteristics of cannabis use in the sample

The characteristics of cannabis use in the sample are summarized in table 3. There were four first time users with psychotic symptoms and two (50%) of them were diagnosed with SSD during follow up. At the time of first admission, a significant percentage (38, 63.3%) had used cannabis for more than 1 year (the duration of use was recorded in only 60 of the 103 patients). The number of short duration users (less than one month) was few (5, 8.3%). Of the patients who reported the frequency of use (67), Forty four (65.6%) were daily users. The patients used two commonly available formulations, cigars and the Madana Modakaya. The latter is a solid pulp of cannabis extract that can be chewed. However, these are illicit cottage preparations and the concentrations of chemical constituents (including that of tetrahydrocannabinol) in this preparation have not been quantified. Fifty nine patients smoked cannabis (57.3%), 21 (20.4%) chewed the Madana Modakaya and 23 (22.3%) used both. Eighteen (17.5%) cannabis users had a family history of a psychiatric illness. Forty three patients (91.5%) started using cannabis before the diagnosis of SSD. Only 3 (6.4%) patients had started using it after the diagnosis of SSD. Twenty four (23.3%) patients in the sample continued to use cannabis after the initial presentation and all of them had psychotic relapses during follow up.

**Table 3:** The characteristics of cannabis use of patients admitting to life time cannabis use (n-103)

Variable	Number	Percentage (%)
**Duration of use**		

1 day	3	2.9

1 week	2	1.9

1-4 weeks	0	0.0

1 - 6 months	11	10.7

7 -12 months	6	5.8

1 - 5 years	20	19.4

More than 5 years	18	17.5

Unknown	43	41.7

**Frequency of use**		

Daily	44	42.7

more than once a week	2	1.9

About once a week	8	7.8

Less than once a week	9	8.7

First time	4	3.9

Unknown	36	35.0

**Most recent use**		

Within 72 hours	41	39.8

3 -7 days	4	3.9

1 -4 weeks	2	1.9

1 -12 months	5	4.9

12 months ago	14	13.6

Unknown	37	35.9

**Formulation of cannabis**		

Cigars	59	57.3

Madana modakaya	21	20.4

Both	23	22.3

On concurrent substance use, alcohol was the most frequently used (54, 52.4%, missing data 1.9%) substance. Nicotine use was also frequent and the formulations included cigars, cigarettes and a local preparation called 'beedi'. Twenty one (20.4%, missing data: 12.6%) cannabis users were using nicotine at time of presentation. Only one person admitted to heroin use.

### Comparison between SSD patients and non SSD patients in the LTC user group

As mentioned before, 47 (45.6%) patients in the LTC user group were diagnosed with a SSD during the follow up. Of the others (56, 54.3%), the diagnoses were as follows; bipolar affective disorder (15, 14.6%), depression (5, 4.9%) and alcohol withdrawal (2, 1.9%). Clinic records also showed that 17(16.5%) patients (10 of them in their first presentation) were diagnosed as CIP. In this group, 7 (41.2%) were diagnosed with SSD during follow up and they were included in the SSD group. In the other ten, the diagnosis remained as CIP. The diagnosis was unclear in another 24 patients (23.3%).

There were no statistically significant differences (assessed with chi square test and Fisher's exact test) in the two groups with regard to age of presentation, marital status, educational level, concurrent alcohol/nicotine use and past consultation at a psychiatry clinic. However, the LTC user SSD patients were more likely to have a positive family history for a psychiatric disorder than non SSD LTC users (RR - 3.1, 95% CI: 1.19 - 8.06). The chi square value for this association was significant (table 4).

**Table 4:** Comparison of patient subgroups within life time cannabis user group on demographic and clinical correlates

Variable	^†^LTC user SSD** patients (n-47) Vs other LTC users (n-56)	CIP^# ^patients (n-17) vs. other LTC users (n-86)	CIP-SSD (n-7) patients Vs. other SSD patients in LTC user group (n-40)
	**Chi square value (degree of freedom - 1)**		

Age at presentation (less than 30 years vs. more than 30 years)	0.254	0.113	1.422

Marital status	1.81	1.2	2.598

Level of education (Secondary education vs. no secondary education)	0.4	0.15	3.065

Concurrent alcohol use	0.002	0.002	1.101

Concurrent nicotine use	0.615	0.0004	2.874

Past psychiatric consultation	0.546	4.22*	9.549*

Family history of a psychotic disorder	5.6*	0.0004	0.953

Subsequent diagnosis of SSD		0.163	

Subsequent diagnosis of bipolar affective disorder		3.48	

Leaving against medical advice	0.054	2.874	0.146

Attending follow up	3.841*	1.428	2.874

Statistical tests used are the chi square test and the Fisher's exact test, *p < 0.05, **SSD- Schizophrenia spectrum disorders, ^†^LTC user - Life Time Cannabis user, ^#^CIP-Cannabis induced psychosis

### Comparison between patients diagnosed as CIP and other LTC users

Seventeen (16.5%) patients in the cannabis using group were diagnosed as having CIP. On comparing the characteristics of this subgroup with the rest of the LTC users, having a past consultation at a psychiatry clinic was the only significant finding (Table 4). There were no significant associations (assessed with chi square test and Fisher's exact test) for age of presentation, marital status, educational level, concurrent alcohol/nicotine use, family history of a psychiatric disorder, subsequent diagnosis of SSD or bipolar affective disorder and compliance with treatment (leaving against medical advice, attendance in follow up clinics).

The mean duration of hospital stay for the CIP group was 9.62 days [standard deviation (SD)-11.4, range 0-33] and for the rest of LTC users it was 17.03 days (SD - 17.5, range 0-107). This difference was not statistically significant (unpaired t test; df-101, t-1.67, p = 0.097). Similarly, the average number of psychotic relapses after the initial admission was 1.62 for the CIP group (SD - 1.62, range 0-6) and 2.01 (SD - 2.39, range 0 - 13) for other LTC users. This was also not statistically significant (unpaired t test; df-101, t-0.64, p = 0.521). We also compared the CIP patients who had a past psychiatric consultation with other CIP patients regarding the duration of hospital stay and relapse rate. Again, there was no significant difference between the two groups (unpaired t test; p = 0.717 and 0.392 respectively).

### Comparison between SSD patients in CIP group (CIP-SSD) and other LTC user SSD patients

This section compares the 7 patients diagnosed with SSD after having an initial diagnosis of CIP with other SSD patients in the LTC user group. The mean duration to diagnosis of SSD since initial presentation was 36 months [SD -41.28] for these 7 patients. The only significant difference between the two groups was that CIP-SSD patients were more likely to have had a past consultation at a psychiatry clinic (Table 4). The age and presence of positive symptoms at admission did not show any difference between CIP-SSD and other LTC user-SSD patients. In both groups, the mean duration of hospital stay was 16 days (SD - 16.8 and 14.3 for CIP-SSD and other LTC user-SSD groups respectively).

## Discussion

### Prevalence of cannabis use

The prevalence of cannabis use in the general population varies according to the acceptance of its use and legal restrictions. The cultivation, use and sale of cannabis (except for medicinal purposes) are illegal in Sri Lanka. The population prevalence of cannabis use in Sri Lanka as estimated by the National Dangerous Drugs Control Board (NDDCB) was 3% in 2003[30]. The global average for cannabis use in adult population is estimated to be around 4%. However in many developed regions, the percentage is above the global average (Oceania -16%, North America - 11%, and Western Europe - 7%)[31]. In our sample which was a hospital based one with psychiatric illnesses, the prevalence of life time use was 2.8%.

Substance abuse, and even alcohol use, is relatively less among women in Sri Lanka, as it is not culturally acceptable per gender norms. The consumption among females contributes considerably to the prevalence of cannabis use in populations of industrialized nations (where it has been estimated) [8,32,33]. In our sample, the cannabis use among females was nil. The very low prevalence of cannabis use in females may contribute to the overall low prevalence in Sri Lanka. In addition, legal restrictions may also limit the accessibility to the drug. However, it should also be noted that the prevalence of substance use is largely under-reported. Due to legal restrictions and social norms, many patients do not reveal the use of cannabis (unfortunately, testing for cannabinoids in urine is not performed in government hospitals due to the expenses incurred).

### Cannabis, SSD and gender

As mentioned previously, many studies published since 1987 have shown an association between cannabis use and SSD[3]. At the same time, the current global epidemiological data indicate that sex ratio of schizophrenia is equal [34,35]. However it is interesting to note that in many studies assessing the prevalence of SSD in substance abuse samples including that of cannabis, males have significantly higher rates of SSD compared to women. Regarding cannabis use, many of the published studies are from industrialized nations. Still it is observed that the number of female cannabis users is less compared to male users (many studies report percentages between 16% - 22% for females in their samples)[6,32,36,37]. Arendt et al[6] who followed up 535 patients treated for first episode psychosis report that male sex and younger age to be risk factors in developing subsequent SSD. It is interesting to note that among females also, the cannabis users were more likely to be diagnosed with SSD than non using females. Mauri et al[37] who assessed the history of substance abuse in first episode schizophrenia patients, report that 80% of the sample had life time cannabis use. The number of male substance abusing schizophrenic patients was significantly more than the number of female patients. Similarly Barnes et al[8] in a study of 152 first episode schizophrenia patients report lifetime substance abuse in 68% of the sample. Cannabis was the most commonly used substance with 64.4% of the total sample having used it. In the overall assessment, there were a greater proportion of males in the substance abuse group compared to non users (76.3% vs. 62%). In another study by Miller et al[38], with 112 first episode schizophrenia patients, 34 admitted to current/previous cannabis use and 30 of them were males.

It is interesting that in almost all of the studies quoted above, male cannabis users had higher rates for subsequent diagnosis of SSD. Yet, apart from Arendt et al[6] other authors have not elaborated on this. Two studies [8,37] have collectively analyzed all substance users together in their gender analysis though cannabis was the most used substance in both samples.

The absence of cannabis using females in our sample is in contrast to many previously mentioned studies. It may be questioned whether women/family would actually reveal cannabis use to a medical doctor given the social stigma involved especially when information gathering is not anonymous. However, several large scale anonymous data collections (by self report) support the observation that substance use (tobacco and alcohol) is very low among the general Sri Lankan female population [39-41]. The global health survey in 2003 estimates that the prevalence of smoking tobacco among females in Sri Lanka to be 2.9%, while the same value for males was 39%[42]. The same survey also showed that life time abstinence for alcohol was 98.3% for women (66.5% for men)[41]. The WHO GENACIS survey in 2002 also showed that a) heavy and hazardous drinking, b) episodic heavy drinking were not reported from females (0%) while the same percentages for men were 15.6% and 13.3% respectively[41]. These observations support our results of a zero self reported cannabis use among females in this sample. Population data of cannabis use among Sri Lankan women is not available but indirect evidence comes from the number of cannabis related arrests in 2003. Out of 9,556 cases, only 3% involved women[43]. 'Involvement' does not necessarily mean consumption and some of these arrests would have been due to drug possession alone.

While assuredly other gender related and social factors may play a role in this, there was nonetheless a statistically significant increase in the prevalence of SSD among men (see above) in our sample. It also showed a strong association between cannabis using males and SSD. The absence of female cannabis users however, has an impact on final conclusions. The logic in that regard is as follows;

1. Overall, in our sample, males were significantly more likely to be diagnosed with SSD than females

2. This finding is not in keeping with the global epidemiological data of equal sex incidence of SSD

3. Cannabis use may be one risk factor for SSD in men as women didn't use it

4. This hypothesis is supported by the similar observations of previous studies cited above

However, what our study cannot exclude is that whether cannabis using females are also at risk of SSD. In fact Arendt et al[6] have indicated the possibility of that being so. The data on relative risk for female cannabis users to develop SSD is limited. Such a comparative analysis is warranted but needs to be carried out in a community with both male and female users.

### Association between cannabis and SSD

Three different hypotheses were mentioned earlier regarding the association between cannabis use and SSD[10].

1. Cannabis use causes SSD (causal model)

2. SSD patients are attracted to cannabis use (self medication hypothesis)

3. Cannabis use predisposes to SSD in a vulnerable minority

On the first theory, it is observed that only a few cannabis users develop SSD. In addition, the incidence of schizophrenia has not increased despite an increase in cannabis use worldwide[44]. Though many studies show that cannabis use precedes SSD, there is no evidence to say that cannabis use per se causes it [3,5,45]. In our sample only 8.1% of patients with SSD had a history of cannabis use and only 45.6% of cannabis users were diagnosed with SSD during the follow up. Arendt et al have reported a percentage of 44.5% in this regard in a similar study[6].

Regarding the second hypothesis, if psychosis prone individuals are attracted to cannabis use, then it is likely that SSD population has more cannabis users. Findings of some studies support this theory [46-48] while others do not[49,50]. Even in studies showing such an association, the temporal relationship is unclear (which started first?). Many demonstrate that cannabis use started before the onset of SSD, but the evidence to contrary is lacking [3,5,45]. Archie et al[32] have actually shown a reduction in drug abuse in a follow up cohort with early intervention. In our sample also, a larger proportion (91.5%) has started using cannabis before the first admission (first recorded episode of psychosis) compared to the 6.4% starting use after the first admission. However 'first admission' may not always be synonymous with 'onset of disease'. It may also be possible that those in a prodrome tried to self medicate themselves with cannabis and presented later. This cannot be clarified due to the retrospective nature of the study.

Many recent advances have been made on the third hypothesis, where it is assumed that a third factor increases the risk for SSD in some cannabis users. This risk factor may be genetic, environmental or a combination of them. Studies on polymorphism of cannabinoid receptor and of the gene encoding the catechol-O-methyl transferase (COMT) enzyme have indicated a possible genetic influence in this regard (see introduction)[11,51,19,53].

However, SSD is not purely a consequence of genetics. The role of environmental factors in constituting a vulnerability to SSD with cannabis use is less explored (see introduction). However, in our subgroup analysis (table 4), there was no difference between the groups on demographic or clinical correlates such as level of education, concurrent substance use, civil status or family history of a psychiatric illness. Having a family history of a psychiatric disorder was significant in the sample when LTC using SSD patients were compared to cannabis using non SSD patients. However, this association is expected given the genetic vulnerability to SSD. Alcohol and Nicotine were the other substances of abuse consumed in this cohort. However, this consumption also did not appear to be significantly different between the subgroups studied. A complete analysis based on the level of income, occupation and living conditions could not be carried out due to incomplete data.

### Cannabis induced psychosis (CIP); is it a 'true' diagnosis?

The diagnosis of CIP has been extensively debated. Many authors are reluctant to accept such a diagnosis on the premise that this 'psychosis' is in fact early onset schizophrenia [27,28]. Still, some hold that such a diagnosis exists[54]. What is consistently shown is that psychotic episodes with cannabis are characterized by predominantly positive symptoms and occur at an earlier age. In our sample, the majority of patients presented with positive symptoms (93.6%). Yet, we could not elicit any significant difference between those having a presentation of CIP and others (or CIP-SSD patients and other LTC user SSD patients) regarding age and positive symptoms.

On comparison of a) CIP patients with other LTC users and b) CIP-SSD patients with other LTC using SSD patients; having a past psychiatric consultation was significantly more for the CIP and CIP-SSD patients. This can be interpreted in two ways. One is that those who are going to develop psychotic manifestations with cannabis are likely to have some background vulnerability. On the other hand it may be so that some of them were in a prodromal stage of SSD to begin with and the onset of illness predated the start of cannabis use. These two issues cannot be reliably verified in a retrospective analysis.

We also could not observe a difference between CIP patients and other LTC users with respect to the duration of initial hospital admission or number of psychotic relapses afterwards. While some authors have observed that CIP patients without a previous psychiatric history to recover faster[55,56], this could not be demonstrated in our sample. However, the numbers in this sample were small for a comprehensive analysis.

On follow up and compliance related matters, previous studies have noted conflicting observations. Some authors report no change in compliance compared to those without cannabis use [32]. Two prospective studies from cohorts in underprivileged communities in New York, USA and Pakistan demonstrate less compliance with treatment among the cannabis users [38,57]. In our sample, loss to follow up was significantly less in LTC user SSD patients when compared to other cannabis users (table 4).

### Policy on cannabis, is a total ban justified?

As mentioned previously, the cultivation, transport, sale and use of cannabis is prohibited by law in Sri Lanka except for medicinal purposes (mostly in oriental Ayurvedic preparations). The prevalence of LTC use in our sample was 2.8%. This was remarkably similar to the estimated population prevalence of cannabis use (3% in 2003 with an estimated 600,000 users)[30]. Cannabis is the only illegal drug that is cultivated within Sri Lanka (mostly in rural areas). Almost all the cannabis intercepted by law enforcement authorities are in the form of unprocessed herb (73,714 kg in 2003)[30]. The small amounts of Hashish or cannabis resin confiscated are believed to be imported. Cannabis related arrests in the time period concerned was 50 per 100,000 and was increasing (29% increase in 2003 compared to 2002). In 2008, 71% of drug related arrests were due to cannabis[58].

The socio demographic pattern of cannabis use is mainly rural and among men. The usual portrayal of cannabis is that of a 'poor man's' drug. Still, it is the cheapest way to get 'drugged'. The 'Madana Modakaya' mentioned previously is easily accessible to many school children and adults and is sold for a paltry sum of Rs.5 (USD 0.05). The average price of 1 kg of Cannabis herb in 2003 was only USD15 (increased to USD 174 by 2008). Other narcotics are considerably more expensive (Street price of 1 kg of opium was USD 5000 in 2003)[30]. The social acceptance of cannabis may also have changed over time. There is an increased glorification of cannabis use ('smoking pot') in media (movies, blogs, songs and social networks) which may influence the young to try out cannabis[59].

To determine whether a total ban is justified, it is important to examine the possible harms and benefits of cannabis use. This has to be balanced against the possible harms and benefits of a ban. There are two schools of thought in this regard. The first is that cannabis use should be legally prohibited as the risks outweigh the benefits. The second view is that cannabis use should not be prohibited. The basis for this argument stems from one or many of the following assumptions;

1. banning would lead to more unlawful use

2. cannabis is a harmless or less harmful drug compared to tobacco and alcohol

3. rather than a total ban, controlled use should be allowed

4. incarceration of mentally ill patients on drug related crimes may worsen their prognosis

5. legalization would lead to less stigma and more importantly it will avoid the unnecessary incarceration of the great majority that uses cannabis without long standing ill effects

Several countries that have lifted legal restrictions on cannabis use (e.g. Mexico, Netherlands). However, none of them have completely legalized cannabis. The term that describes the legal status of cannabis in many of these countries is 'decriminalization' or 'depenalization' which refers to a reduction or non enforcement of laws prohibiting cannabis use though it is not legally accepted[60]. The legal status of cannabis is complex in some countries where it's decriminalized. For example in Netherlands, sale and possession of cannabis is illegal while personal use is allowed[61]. 'Legalization' refers to a more 'liberal' state where government may enforce some restrictions on specific activities such as advertising and sale, but largely 'accepts' the use of a drug (e.g. - legal status of tobacco).

It is difficult to establish whether banning cannabis would lead to increased illegal use. The overall low percentage of adult cannabis use (cf. tobacco and alcohol) may be due to the legal ban in many countries and the social stigma associated with it. On the other hand, decriminalization has shown to increase the use of cannabis in several countries, though the causative role in decriminalization is debated [60]. In Southern states of Australia, cannabis consumption increased after penalties were reduced in 1987 but this was not statistically significant compared to other states where use was still illegal[62]. Following decriminalization, cannabis use in Netherlands increased steadily between 1984 and 1992. In other European states and USA, where cannabis use was illegal, the rates did not increase[60]. However after 1992, the rates increased in these countries too and it may partly be attributable to the media influence and increased drug trafficking. The experience in Mexico shows that the area of cannabis cultivation in 2008 had increased by a massive 35% compared to that of 2007[63]. The number of drug related arrests reached an all time high in 2009 though it is not clear how many of these were cannabis related. An increase in cannabis cultivation also affects neighbouring countries as some amount of this harvest will be smuggled across the borders[63].

As mentioned above, another argument of proponents for legalization is that cannabis is a harmless or a less harmful drug compared to tobacco or alcohol. While it may indeed be less harmful than tobacco and alcohol, the notion that it is harmless is unacceptable. As mentioned previously, the impact of cannabinoids on neurotransmitter systems has been demonstrated experimentally. These studies have also demonstrated many short term ill effects of cannabis use such as mood changes, lapses in judgment, short term memory loss and inducement of positive and negative symptoms of schizophrenia. In addition, there is strong epidemiological evidence including that of this study, for an association between cannabis use and SSD. In addition, the hospital based studies only assess the full blown psychotic episodes associated with cannabis use. The more subtle psychiatric manifestations of long term cannabis use may go unnoticed in the population but may contribute significantly to impaired performance and cognition at an individual level. The repeated findings of these experimental, biochemical, epidemiological and genetic studies do not support the view that cannabis is a harmless drug.

However the next question is, whether our response to cannabis use is doing more harm than cannabis itself? While the possibility remains that cannabis may predispose to a long standing psychiatric illness such as schizophrenia, it is also clear that only a few cannabis users are affected to this extent and many use the drug without any long standing ill effects. In our sample, of all cannabis users, only 47 were diagnosed with SSD. This value is further reduced when background risk for SSD is accounted for (as calculated from SSD percentage in non cannabis using patients).

One may argue whether a total ban is justified when

1. Only a small proportion of users are affected

2. A clear aetiological mechanism is yet to be demonstrated

This issue brings forth one of the strongest arguments of proponents for lifting a ban. In countries where cannabis use is totally prohibited as in Sri Lanka, cannabis users; both healthy and mentally ill are imprisoned on drug related charges. Ill effects of this can be assessed in two ways.

Firstly, incarceration of mentally ill leads to a poor patient outcome as the required standard of care including proper treatment, rehabilitation and socializing opportunities are denied to the patient. Though Sri Lanka as many other countries, have mechanisms to prevent unnecessary incarceration of mentally ill individuals, they are not foolproof. Even in United States it is estimated that up to 12% of inmates are having mental health concerns. Some argue that the figure may be even as high as 34% [64]. Despite the numbers being not available for Sri Lankan jails, the strong possibility of mentally ill patients needing inpatient psychiatric treatment being held at prisons on cannabis related charges, have to be accepted.

Secondly, the negative aspect of a legal ban is very significant when considering the impact on healthy individuals (who may use cannabis without any long term ill effects) imprisoned on cannabis related charges. As mentioned previously, the majority of drug related arrests in Sri Lanka are due to cannabis. In USA, drug related arrests reached a record high of 1.8 million in 2005. Marijuana offences accounted for 42.6% of it [65]. The question here is, since those at risk of getting CIP or SSD with cannabis are few, is it justified to incarcerate the vast majority of people who may use cannabis without any harm?. As much as mental illness is associated with stigma, imprisonment is also associated with similar stigma. The ill effects on personal, family and social life are also similar or worse. Hypothetically, if cannabis was legalized, all these arrests and their negative impact would be avoided.

On the other hand, one strong point for the opponents for legalization is that, if a blanket ban is lifted, cannabis use in Sri Lanka has all the right components to grow in to a problematic drug use (It is locally grown, easily accessible, cheap, glorified in the media plus a large cannabis naïve population including almost all the females). It can be questioned whether testing the grounds by legalizing a potentially harmful drug is warranted when we are still trying to tackle the social, health and economic problems of the already legalized tobacco and alcohol.

We would not make an opinion on banning or legalizing cannabis on this paper. While our study shows that a minority of cannabis users are at risk of SSD or CIP, it is difficult to weigh it against the mass, non specific incarceration of potentially healthy individuals (who might use it without any ill effects) on cannabis related offences. There are many loopholes in the current knowledge on cannabis and its ill effects to make a firm opinion in this regard.

What we do suggest therefore, is a more scientific approach to the problem. The ideal situation would be to identify those at risk of getting psychosis and discourage use within this subgroup while avoiding unnecessary and potentially harmful incarceration of healthy and mentally ill patients on cannabis related charges. A good way forward is to make a policy decision on a programme that would address this issue comprehensively on scientific evidence. Such a program should include;

1. monitoring the changing trends of cannabis use in the population

2. measuring the subtle psychiatric manifestations of cannabis use to accurately quantify the substance use burden

3. more funding for studies to identify vulnerability to cannabis related psychotic manifestations

4. counteracting the media glorification of cannabis use

5. curtailing local production of cannabis by education and creating alternate employment opportunities to rural communities dependent on cannabis cultivation (rather than by punitive measures)

6. improving facilities of hospitals to provide testing for cannabinoids so that diagnosis can be confirmed for individuals and epidemiological data can be gathered more accurately for the population

7. a mechanism to recommend necessary changes to legislature on the legal status of cannabis on the available valid scientific evidence

### Limitations

Given the retrospective nature of the study, several limitations have to be noted. The prevalence of cannabis use among patients may be under-reported due to a) concealment by patient b) not being queried by the interviewer c) inability to test for urinary cannabinoids in government hospitals and d) loss of records. Many clinically relevant variables such as family income, occupation, past medical history, living circumstances could not be analyzed properly due to incomplete records. Though cannabis use was exclusive to males in this sample, it is also possible that females with cannabis use were overlooked due to concealment by the patient or failure to elicit a proper history by the interviewer.

There were few other undiagnosed patients with psychotic episodes where the presentation was circumstantially linked to cannabis use. We looked at three factors to establish a link in this regard; duration of cannabis use less than 6 months before the first admission; more than once weekly use and use within last 72 hours of admission. On these criteria, a further 16 (15.5%) patients had their symptoms circumstantially linked to cannabis. This group was not included in the analysis as the confounding factors for such a presentation cannot be established from records alone.

The diagnostic criterion used in many studies quoted in text is the diagnostic and statistics manual (DSM - IV). However psychiatric morbidities in Sri Lankan hospitals are diagnosed with ICD - 10. This may have an impact on head to head comparisons of our results and others. For example, a diagnosis of schizophrenia requires a minimum period of 6 months with symptoms in DSM IV while it is just one month in ICD 10. This may result in a larger number of formal diagnoses with ICD 10.

As discussed previously, difficulty in identifying the actual onset of symptoms/illness by retrospective examination is also a limitation of the study. It should be further noted that though all males and females admitted and/or consulted in clinics during the period of study were compared with each other with respect to SSD, these two groups may not be matched with relation to age, family history, co morbidities and other confounding factors.

## Conclusions

In this retrospective cohort with psychiatric illnesses, male sex and LTC use were significant risk factors for SSD. Majority had used cannabis before the diagnosis of SSD. However we could not identify a particular subgroup of users that are at increased risk to recommend targeted primary prophylaxis. However, this vulnerable group may be over represented in populations with already diagnosed psychiatric morbidity and therefore cannabis use may be discouraged in such groups.

In our opinion, further research on cannabis use in Sri Lanka can develop in three overlapping spheres.

Firstly, there is the need for hospital based studies on psychiatrically ill populations to clarify several issues that we could not address given the retrospective nature of our study. These include; the establishment of timeline between start of symptoms and cannabis use (to exclude start of use during a prodrome), use of laboratory confirmation of recent cannabis use to improve accuracy of data, correlating symptom severity with the amount of herbal cannabis consumed, exploring a family history of SSD and cannabis use to identify a genetic vulnerability for both behaviours and assessing efficacy of different pharmacological therapies to treat CIP or SSD following cannabis use. These issues can be addressed by prospective cohort studies or by randomized controlled clinical trials (with regard to efficacy of pharmacological therapies).

Secondly, it is important to conduct community based studies to assess the prevalence of cannabis use and subtle psychiatric morbidity of this use that may not present to hospitals for treatment. One possibility is to follow up a large birth cohort and see the impact over the years. Needless to say it is a very costly and arduous exercise. Another possibility is to identify 'psychosis prone' individuals through screening tools and follow up such cohorts. Both types of studies can be found in literature but has not been conducted in populations of developing nations where the social dynamics are different.

Thirdly, there is the need for more experimental and empirical laboratory studies for purposes of quantifying the cannabinoid compounds in herbal cannabis and in local preparations as 'madana modakaya'. As mentioned previously, the cocktail of chemicals in herbal cannabis contain chemicals with contrasting effects. The relative concentrations of each may also contribute to the nature of observed symptoms and their severity.

## Abbreviations

Abbreviations are explained where they are first used in the text.

## Competing interests

The authors declare that they have no competing interests.

## Authors' contributions

All authors have contributed to the concept and design of this study, writing the manuscript and approving the final draft.

## Author information

CR (MBBS) and SW (MBBS) are medical officers of Mental Health in Psychiatry unit, Provincial General Hospital, Ratnapura, Sri Lanka. AG is a nursing officer in psychiatry and GJ (MBBS, MD) is the Consultant Psychiatrist of the unit. CM (MBBS) is a Research Associate of the Department of Clinical Medicine, Faculty of Medicine, University of Colombo while SR (MBBS, MD, MRCP) is the Head and Senior Lecturer in the same department.
